# Normal Trauma and Abnormal Diagnosis: A Discursive Analysis of Sexual Violence Law and Policy in England and Wales

**DOI:** 10.1007/s10691-025-09588-x

**Published:** 2025-11-06

**Authors:** Emma Yapp

**Affiliations:** Faculty of Humanities and Social Sciences, Birkbeck College, University of London, 26 Russell Square, London, WC1B 5DQ UK

**Keywords:** Sexual violence, Rape, England and Wales, Trauma, Mental health, (ab)normal

## Abstract

This paper interrogates the discursive construction of the relationship between sexual violence and ‘mental health’ in case law and legal policies from England and Wales. It shows that the law invariably enacts a binary discursive construction of those who have experienced sexual violence where mental health evidence is introduced. Individuals are constructed as either experiencing ‘normal trauma’ or an ‘abnormal diagnosis’. These findings are contextualised within recent feminist legal reforms to the treatment of mental health evidence in sexual violence cases, and emphasise the pressing need to centre the experiences of those who identify with psychiatric diagnoses in feminist anti-sexual violence politics.

## Introduction

Experiences of sexual violence are now largely inseparable from the language of ‘trauma’ in the popular imaginary (Gavey and Schmidt 2011; Marecek 1999). This expectation is a normative one, such that individuals may struggle to garner legitimacy if they do not articulate their experiences of sexual violence in the expected (traumatised) way. Judgments of the legitimacy of experiences of sexual violence are additionally impacted by the discursive legacies of gender, racial politics, and class; particularly within the courtroom setting (Russell 2013, 2016; Larcombe 2002a, b; Hengehold 2000; Crenshaw 1990; Powell et al. 2017). However, to date, scant attention within either feminist or socio-legal scholarship has been afforded to the discursive relationship between sexual violence, trauma, and mental health evidence more broadly. Commentators such as Alyson Spurgas have suggested that the ‘ideal’ sexual violence victim in the popular imaginary is characterised by not just being white, middle-class, and subject to a ‘real rape’, but additionally: being psychologically ‘normal’ beforehand (Spurgas 2021). This suggestion raises questions around how experiences of sexual violence are adjudicated in relation to understandings of ‘mental health’, particularly within the legal setting. The law is a powerful arbiter in the adjudication of sexual violence, and holds symbolic influence for how sexual violence is understood and evaluated more generally.

This article accordingly examines legal judgments and associated legal policies pertaining to sexual violence and mental health evidence in England and Wales. It argues that when mental health evidence is introduced in sexual violence trials, individuals are invariably discursively constructed in one of two ways: normal and traumatised, or abnormal and unreliable. This is significant as it provides further evidence for the narrow and exclusionary parameters around judgments of experiences of sexual violence, and in particular, how those who seek mental health support may struggle to garner legitimacy for their experiences. The discursive construction of mental health evidence additionally reflects a legal conception of sexual violence as rendering a bodymind incapacitated (Bourke 2012).^1^ Further, ‘normal trauma’ acts as ‘proof’ of violence, while ‘abnormal diagnosis’ functions as a ‘prism’ of distortion and unreliability. Before moving to explicate the methodological approach and analysis in full, it is first useful to offer an account of the role of mental health evidence in feminist engagements with sexual violence law and policy reform in England and Wales.

## Mental Health Evidence and Sexual Violence

Feminist legal research has shown that mental health evidence concerning someone who has experienced sexual violence has a negative impact on criminal justice outcomes. The presence of psychiatric diagnoses significantly affects police decisions to prosecute and charge suspects in England and Wales (Hohl and Stanko 2015; Ellison et al. 2015), and impacts attrition throughout the criminal justice system (Hester and Lilley 2018; Walker et al. 2021). The psychological scrutiny of those who testify to sexual violence has a well-documented medico-legal history in feminist scholarship (Ellison 2005, 2009; Bourke 2012), and is often traced back to problematic assertions from patriarchal legal forefathers (e.g. Hale 1736), and biases in legal discourses concerning gender (Russell 2013, 2016; Larcombe 2002a, 2002b). Scholars working on jurisdictions outside of England and Wales have additionally suggested that legislative definitions of sexual violence, and increasing restrictions around the use of sexual history evidence, have led to an increase in the use of evidence concerning an individual’s state of ‘mind’ to impugn the credibility of sexual violence testimony (du Toit 2009; Bronitt and McSherry 1997). This context has led legal scholars such as Louise Ellison to call for restricting the admissibility of mental health evidence in sexual violence trials in England and Wales, objecting to the legal treatment of those who speak about violence as lying, hysterical, or ‘mad’ (Ellison 2009).

However, the rise in the trauma paradigm for understanding experiences of sexual violence, and associated diagnoses such as ‘post-traumatic stress disorder’, has resulted in complex legal debates around what kinds of mental health evidence should be admissible in sexual violence trials (Rumney and Taylor 2002; Ward 2009; Summerfield 2001; Ellison 2005; Raitt and Zeedyk 1997; Raitt and Zeedyk, 2003). In 2006, the Government held a consultation on whether expert evidence about the psychological impact of sexual violence should be admissible (Home Office and Office for Criminal Justice Reform 2006). Ellison argued that it should be on the grounds that ‘trauma’ responses are not well understood by the general public (Ellison 2005, p. 264), although the Government decided against this in 2007 (Criminal Justice System 2007; Ormerod 2006; Ward 2009). However, today, judges are able to direct the jury on the nature of psychological trauma following sexual assault (Maddison et al. 2020; s20), and evidence of ‘psychological injury’ such as PTSD is theoretically admissible in criminal trials, as long as the evidence speaks to the nature of the diagnosis in general, rather than the credibility of individual testimony in particular (Crown Prosecution Service 2021).

Further, the year 2024 brought a significant feminist legal “campaign win” (Rape Crisis England & Wales 2024; EVAW 2024) regarding mental health evidence in sexual violence trials. An amendment to the Victims and Prisoners Bill aims to restrict the ‘relevance’ of any mental health evidence accrued pre-trial to sexual violence cases, in an effort to curtail its impact on credibility (UK Government 2024).^2^ This reform came, in part, in response to public outcry at an outdated legal policy from 2002 that recommended people should not discuss their assault in therapy pre-trial (Kale 2019; Chakrabarti 2019; Crown Prosecution Service 2002). The policy was perhaps intended to prevent therapeutic records being used to establish inconsistencies or unreliability in individual accounts at trial, but raised additional concerns for anyone who had accrued mental health records prior to an assault. The media attention led to a significant overhaul of legal policies on sexual violence and mental health evidence between 2019 and 2022 (e.g. Crown Prosecution Service 2019; 2022). This included both new prosecutorial guidance on the provision of pre-trial therapy (Crown Prosecution Service 2022), and on mental health evidence in sexual violence cases more broadly, which aimed to facilitate “understanding of pre-existing mental ill health, and potential psychological reactions to sexual assault and its aftermath” (Crown Prosecution Service 2019, 1).

The narrative laid out thus far is just one of the stories that we can tell about feminism, sexual violence law and policy reform, and the role of mental health evidence in England and Wales. It has a clear narrative of ‘progress’ (Hemmings 2011), culminating in the recent ‘victory’ of legal reforms to the ‘relevance’ of mental health evidence in sexual violence trials. However, Joanne Conaghan and Yvette Russell recently offered us a pertinent reminder that when attending to the discursive in sexual violence law, neither ‘evidence’ nor ‘relevance’ operate as neutral concepts (Conaghan and Russell 2023). In the latest reform to the Victims and Prisoners Bill, as with Ellison’s work, it is suggested that mental health evidence should only be adduced where ‘relevant’ to credibility (UK Government 2024; Ellison 2009). For Ellison, evidence of psychological trauma is relevant (Ellison 2005), as well as evidence demonstrating that a person’s ‘capacity or disposition to provide reliable evidence is negatively affected by a mental illness or disorder’ (Ellison 2009, p. 43). Ellison thus assumes a positivist approach to mental health: that psychiatric diagnoses, and their relationship to the ‘reliability’ of an account of sexual violence, are empirically verifiable, rather than deeply mired in histories of racism, classism, ableism, and other dynamics of power (Goozee 2021; Spurgas 2021; Szasz 2007; Ussher 1991; Mollow 2006). While feminist legal scholarship, and the associated legal and policy reforms, have been an important facet of feminist anti-violence politics, this representation of mental health largely neglects how socio-cultural ideas about gender, race, class, sexuality, and (dis)ability come to bear on the adjudication of both sexual violence and mental health. Rather than asking whether mental health evidence is relevant in sexual violence trials, this paper asks how the relationship between sexual violence and mental health has been discursively constructed over time in law and policy.

### Method

The data for this paper consists of both legal judgments (*n* = 13) and associated policy materials (*n* = 8). Both civil and criminal judgments were included due to the higher level of detail in the former, as well as legal policy materials pertaining to adulthood sexual violence and either trauma or mental health evidence. Civil and criminal cases use different standards of proof, as well as different general procedures, but given that I exclusively obtained cases in the form of judgments, analysing both was fruitful for insight into the way these people are evaluated, adjudicated, and discursively constructed. Cases were identified through keyword searches of relevant databases (WestLaw, LexisNexis, and Bailii) and citation tracking; a twenty-year period was examined (2002–2022). Cases were included where full judgments were available, and where those judgments explicated either general ‘trauma’ or ‘mental health’ evidence in relation to an experience of sexual violence occurring on or after age 16. Cases pertaining to children or people with intellectual (dis)abilities were not included as these are governed by different legislative and discursive frameworks (cf. Benedet and Grant 2014; Bourke 2020), although some were additionally consulted for context (*n* = 6).^3^ Policy materials were identified using the 2002 guidance on pre-trial therapy as a starting point, as well as the associated policy updates between 2019 and 2022. Acquisition of materials concluded in June 2022. Materials were collated and managed in NVivo.

Materials were analysed discursively, attending to how the relationship between sexual violence and mental health evidence is constructed by law. Analytical primacy is given to the case law materials in this article, although additional consulted cases and policy materials are brought into the discussion to shed light on the wider legal treatment of sexual violence and mental health. The discursive analysis is informed by the theorising of Michel Foucault (Foucault 1969, 1977), particularly its application in feminist empirical work (Pillow 2003; Phipps 2010; Ussher and Perz 2014). First and foremost, this theoretical approach notes the centrality of dynamics of power in shaping discourse, knowledge, and ultimately experiences; giving analytical primacy to the roles of race, gender, and sexuality within this. For this paper, the role of (dis)ability is also central. Here, (dis)ability signals experiences of mental distress currently organised as ‘mental health’ problems or psychiatric diagnoses (Johnson 2021; Carter 2021). The use of the parenthetical follows Sami Schalk, aiming to both take seriously the lived experience of those who identify with psychiatric diagnoses, and how these categories are shaped by bodily and mental ‘norms’, as well as their broader political and socio-cultural context (Schalk 2018). It should, however, be noted here that demographic information is generally not available in the legal judgments assembled for this article. Information about general ‘mental health’ and psychiatric diagnoses is more salient, but it is important to state that this is not reflective of individual identification with particular diagnoses, but externally designated, and legally constructed. Findings are thus contextualised in appropriate literature throughout, but not indicative of empirical evidence of individual experiences.

## Findings

Two discursive constructions were identified in the analysis: ‘normal trauma’ and ‘abnormal diagnosis’. Much of the aforementioned legal debates concerning mental health evidence in sexual violence trials are traced back to one case that makes a distinction between “ordinary folk” and those suffering from “mental illness”.^4^ This implicit binary distinction between a ‘normal’ and ‘abnormal’ bodymind is powerfully evident in the two discursive constructions identified here. The analysis establishes a remarkable consistency in the two identified discursive constructions over the time period. In examining the function of these constructions in terms of their impact on adjudicating the ‘truth’ or ‘falsity’ of violence, several other facets were identified. All ‘successful’ cases were characterised by either the specific feature of a ‘freeze’ response to sexual violence (*n* = 3) or of normal trauma more generally (*n* = 4). While this observation is not intended to establish any kind of causal connection between evidence and judgments, it does show that the *only* form of mental health evidence that is seen as legitimate in the context of sexual violence trials is, specifically, diagnoses or symptoms associated with ‘trauma’. Further, the way in which these two discursive constructions function additionally betrays an inherent legal scepticism of the relationship between ‘mental health’, memory, and sexual violence. This latter point will be fleshed out in more detail after discussing the construction of normal trauma and abnormal diagnosis in turn.

### Normal Trauma

In this section I detail the discursive construction of ‘normal trauma’ in the materials. Even though evidence of PTSD as ‘psychological injury’ was deemed admissible in criminal trials in 2011, no subsequent cases were identified in which this was the case; solely one civil case.^5^ The freeze response is a notable characteristic of the discursive construction of ‘normal trauma’ for three reasons. It contributes to legitimacy in the ‘success’ of these cases; it functions as physically instantiated diagnostic evidence or ‘proof’ of violence; and is reflective of historical British medico-legal understandings of sexual violence causing incapacitation, or ‘insensibility’, thus effectively removing the question of consent (Bourke 2012).

## Freeze Response

On the same day that the consultation on the admissibility of ‘trauma’ evidence in sexual violence trials was opened to the public in 2006 (Home Office and Office for Criminal Justice Reform 2007), the Court of Appeal sat to adjudicate the appeal of a psychiatrist, Christopher Allison, who had been convicted for multiple counts of rape and sexual assault against his patients. Allison appealed against his conviction largely on the basis that his wife had since found a letter from one of the people he assaulted expressing sorrow at his professional suspension after the allegations came to light, and that this behaviour was “implausible” from a woman who claimed to have been raped.^6^ It was also argued that this woman returning to therapy with him after the first sexual assault was ‘abnormal’ behaviour for someone who had experienced sexual violence. This, coupled with Allison’s own psychiatric notes describing her as “whorish”, and his account of how she “had submitted to sex willingly”, was clearly intended to rewrite this woman’s account of sexual violence into one of objective consensual sex.^7^^,^ 8

However, in the dismissal of Allison’s appeal, the judge reiterated the woman’s own descriptions of her trauma, in her use of the language of ‘freezing’. Her voice is re-animated in the judgment to describe “how your body just stays there and you are furious with your body because you can’t just get up and go”.^9^ In another judgment, it states that the woman “repeatedly told him to stop, but that she felt like “a rag doll” and was unable to move a muscle in her body or offer any resistance”.^10^ Judith Herman’s (1992) foundational conception of the ‘freeze’ response to sexual violence similarly quoted people feeling like a “rag doll” (Herman 1992, 42), and this language was gaining traction in medical jurisprudence around the time of Allison’s appeal. In 2007, Fiona Mason, a forensic psychiatrist who had authored training materials on sexual trauma for judges that were being used in 2008 similarly co-authored an article published in the British Medical Journal detailing the health consequences of sexual violence, including being “paralysed with fear and powerless to fight back” (Welch and Mason 2007).^11^ Mason’s influence over legal practice is enduring, as she also co-wrote the currently operational guidance on psychological evidence (Crown Prosecution Service 2019).

Further to assertions that evidence of ‘trauma’ has become too closely associated with ‘proof’ of violence (Gavey 2005; Sweet 2021), the ‘freeze’ response is additionally characterised as medically diagnosable. For example, in *R v Soroya*, a young Polish woman had arrived in the UK without a work permit and needed a way to make money while she waited to accrue one.^12^ She responded to an advert in a Polish newspaper for cleaning work, which is how she met Soroya, who was subsequently convicted of raping her when she went to collect her first round of wages. In the appellate judgment, it states that “She was not physically injured or threatened and needed to explain why she had put up no obvious resistance”.^13^ In the original trial, the reason for her lack of “obvious resistance” was attributed to a diagnosis of a “conversion disorder” adduced by the prosecution, which meant that she “might be abnormally passive in the face of stressful circumstances”.^14^ Although no longer a psychiatric category in the international diagnostic manual, a “conversion disorder” was previously categorised under “dissociative disorders”, “being associated closely in time with traumatic events” (World Health Organization 1992; F44.4); a kind of ‘trauma’ diagnosis. The conclusion was to the effect that she was “not able to fight back as she froze due to psychiatric illness”.^15^ Accordingly, it was decided that she was not able to enact ‘resistance’ due to a trauma-induced “illness”; understandings of trauma, and the associated ‘freeze’ response here function as evidence of violence.

The ‘freeze’ response is especially persuasive to the trial setting on account of it being physically instantiated, and hence, ‘proof’ of trauma and violence. In the legal treatment of gendered violence, there is an overemphasis on physical and bodily ‘injuries’ (Bourke 2012; Sweet 2021, 183). This construction of ‘normal trauma’ is remarkably reminiscent of the British medico-legal notion of ‘insensibility’. Historian Joanna Bourke has demonstrated that before the language of psychological trauma was applied to sexual violence in the UK, people were considered to be rendered physically ‘insensible’ and incapacitated in psychiatric and legal texts from the 19th and early 20th century (Bourke 2012). Bourke additionally shows that adjudicating experiences of sexual violence as resulting in ‘insensibility’ actually served to *legitimate* individual experiences of sexual violence. She writes that “the ‘sensible’ body was seductive and either invited abuse or would have been able to repulse any attack. The ‘insensible’ rape victim testified to ‘true’ violation” (Bourke 2012, 33). This notion is evident in Soroya’s case, as the woman’s lack of “obvious resistance” is explained through the idea that she was “abnormally passive”, and “froze due to psychiatric illness”; this traumatic “illness” becomes representative of true violation.^16^^,^ 17^,^ 18 This is also evident in the other ‘freezing’ descriptions being particularly physical and bodily, and framed around an ability to ‘resist’ or not: “not able to fight back”; “unable to move a muscle in her body or offer any resistance”; “you can’t just get up and go”.^19^^,^ 20^,^ 21 The frozen bodymind testifies to ‘true’ violation, in the same way that the insensible one did before it: consent is here deemed medically, or psychologically, impossible.

## Normal before and Traumatised after

In several cases, the judgments draw attention to the contrast in individuals’ lives before and after their assaults, and how they were normal before, but psychologically damaged afterwards.^22^ Here, ‘normal’ before is influenced by ideas about class, as well as being particularly characterised by being specifically *psychologically* normal before an experience of sexual violence (Spurgas 2021). In one civil case, two women were claiming against the police for their mishandling of the John Worboys case – the prolific taxi driver rapist convicted in 2009. In the judgment, one of the women is deemed of “good character”, which is the assessment of the expert psychiatrist.^23^ The judgment additionally reanimates his statement that “she was a normal and confident young woman before the assaults”.^24^ The subsequent damage of the sexual violence is articulated in terms of productivity, and as specifically “damag[ing] her performance at university”, reflecting a potentially high level of education.^25^ Although it is impossible to adduce information specifically about this person’s class from this case, the ‘damage’ being framed in terms of university performance speaks to other literature which demonstrates that popular understandings of sexual violence are additionally constructed around a (white) middle-class subject (Spurgas 2021; Phipps 2020; Serisier 2018; Davis 1981, 2019).

Further, the judgment explicitly connects her being “normal and confident before the assaults” to the fact that “she would not have developed mental health problems had it not been for the assaults”.^26^ The assuredness that she would not have “developed mental health problems” could potentially be partly connected to an assessment of class or material privilege, but it certainly reflects that she was considered not just ‘normal’, but specifically, *psychologically* ‘normal’ before the assault, which then provides further evidence for the ‘traumatic’ effect of her experience. This raises questions around who will be adjudicated as experiencing ‘normal trauma’, and the exclusionary nature of these judgments: is someone with a pre-existing identification with a psychiatric diagnosis less likely to be adjudicated as traumatised? By extension, this judgment suggests that any evidence of psychological ‘abnormality’ before an assault, and therefore any mental health evidence accrued pre-trial, could be considered relevant to claims of sexual violence, and it is to the ’abnormal’ discursive construction that I now turn.

### Abnormal Diagnosis

The discursive construction of an ‘abnormal diagnosis’ consistently represented the implicit alternative hypothesis in these judgments in which they ask, is someone ‘normal’ and traumatised, or ‘abnormal’? At times, this weighing up of the two options was particularly explicit. For example, in *London Borough of Haringey v FZO*, much of the discussion centred around the conflicting assessments of two expert psychiatrists, and whether the man in question was deemed to suffer from ‘emotionally unstable personality disorder’ or ‘complex post-traumatic stress disorder’.^27^ Concern was expressed that this man’s diagnoses only changed to trauma-related categories after he disclosed the abuse, the suggestion explicitly that he had persuaded the clinicians at his private healthcare institution “to change their diagnosis from personality disorder to trauma in order that the health insurer would meet his claim”.^28^ The notion of people essentially ‘faking’ trauma for financial compensation has a long medico-legal history in the UK (Smith 2011; Summerfield 2001). Yet this case is especially notable for the legal reliance on mental health evidence as ‘proof’: either this man has “complex post-traumatic stress disorder”, which provides proof of violence, or “emotionally unstable personality disorder”, which constitutes proof of falsity and manipulation.

There are two other particularly striking features of the discursive construction of an abnormal diagnosis. First, it mirrors the discursive construction of ‘normal’ trauma: where an ‘insensible’ bodymind testifies to true violation, a ‘sensible’ one becomes indicative of falsity. And second, it demonstrates the power and authority of mental health evidence in determining judgments, as indicated by the case above. Both of these points can be usefully illustrated with reference to one particularly detailed case, which requires a somewhat lengthy explication before making each of these points more fully.

Edward Gabbai had initially been convicted for sexual violence against multiple people in 2018. Part of the basis for his appeal in 2019, and the one that ultimately quashed his original sentence, entailed accessing ‘fresh evidence’ in the form of extensive mental health records pertaining to one of the women he had assaulted, all accrued before she had even met Gabbai in December of 2016. In 2014, she saw a university counsellor and discussed one of her prior assaults; the counselling notes are reiterated in the judgement, where they are quoted as saying “I took him back to my flat. I didn’t say no to begin with. Lying. Attention seeker”.^29^ The judgement states that “The implication of the last phrases is, or may be, that they were self-descriptions”.^30^ Given the limitations of analysing judgments, and especially their reproduction of counselling notes from years earlier, it is impossible to know whether this was the case, although it seems unlikely that anyone would describe themselves in this way. On the 12th of October 2016 this woman had an hour-long call with a counsellor at the Maytree Clinic, which is additionally documented as

Raped three times. ‘I put myself in dangerous situations’.

1. 18 years taken from a bar. I only said no half-way through.
2. A guy forced himself on me.
3. In Spain. Maybe wasn’t rape.^31^

On the 19th of October, in her pre-admission notes she is documented as irresponsible (and hence responsible for her experiences of violence): “Has suffered three rapes. Putting herself in dangerous situations (i.e. voluntarily)”.^32^ After a counselling session post-admission, on the 21 st of October, the notes say that she “touched on whether the sexual acting out was more a symptom of her self-hatred. A form of self-harm”.^33^ Then on the 1 st of November 2016, she again clearly reports that she was raped three times during a psychiatric assessment, but the psychiatrist notes that she “tells me that she is always putting herself in danger, found it hard to say no, but there were lots of other times she didn’t [say no] when she wanted to say no”.^34^ The judgment concludes that “In our view, this evidence was of a striking nature, and relevant as suggestive of previous false accounts. The evidence that the complainant had doubted her own past suggestions of rape, and was ‘Lying. Attention seeker’ should have been admitted”.^35^ The notes were admissible as evidence of this woman’s “bad character”: she receives an ‘abnormal diagnosis’.^36^

The conclusion of this judgment is important for two reasons. Firstly, the discussion of this woman’s “sexual […] self-harm” speaks to the opposite side of the construction discussed earlier around individual bodyminds being rendered ‘insensible’.^37^ Where an ‘insensible” bodymind testifies to true violation, this woman’s ‘sensible’ bodymind is constructed as “seductive and either invited abuse or would have been able to repulse any attack” (Bourke 2012, 33). She is the one who is constructed as putting “herself in danger” and inviting abuse, failing to resist the attacks because “she found it hard to say no”; there is no acknowledgement that it could be true both that she often found herself in these situations, and that the assailants were still responsible for sexual violence. This notion of the ‘sensible’ bodymind either inviting abuse or being able to resist it is particularly prominent in contemporary legal and policy discourses (Stringer 2013; Phipps 2010). Lise Gotell, writing of the Canadian legal context, has suggested that legal understandings of ‘consent’ are embroiled in wider stereotypes about respectable (and hence resistant) femininity. She writes that “sex outside, sex that is risky, sex that defies standards of responsibility, respectability and sexual safekeeping, marks the complainant herself as a deviant” (Gotell 2008, 887). In *R v Gabbai*, this woman is adjudicated as failing in effective “sexual safekeeping”, on account of her ‘abnormal diagnosis’ in the form of “self-harm” and “bad character”, which instead diminishes her consent in the same way that Gotell describes.^38^ This lies in stark contrast to the earlier discussion of the insensible bodymind, unable to give consent.

Secondly, the conclusion that this evidence is “suggestive of previous false accounts” and hence admissible, is apparently more persuasive than the judgment’s reproduction of some video evidence of Gabbai’s sexual violence, demonstrating the woman’s explicit non-consent. Extracts from the transcript are repeated in the judgment in which she is clearly depicted as “crying” and saying “No”.^39^ I was able to gain access to the judge’s summing up in the original Gabbai trial, where she is again quoted as saying “I screamed, I shouted, I said no, I cried genuine tears. I was really scared. I thought I could die”.^40^ Yet, despite her “genuine tears” and fears for her life, the idea that she had made previous ‘false’ accusations somehow overruled this evidence of explicit non-consent. Linguistic scholar Susan Ehrlich has noted that video evidence is particularly persuasive to juries (Ehrlich 2018), but it was additionally noted by the judge’s summing up in the original trial that the jury had no medical or mental health evidence to ‘corroborate’ the story.^41^ While again, it is impossible to say from these materials which pieces of evidence were compelling for the jury at the original trial, the mental health evidence became crucial for the judiciary: so crucial that Gabbai is adjudicated as having a reasonable belief in consent while a woman is clearly recorded as ‘crying’. Mental health records here become a way of officiating a counter-narrative of consent, a process described by Alcoff and Gray as occurring when “the disclosure and speaking out by victims of sexual violence is transformed into evidence of their own pathology” (Alcoff and Gray 1993, 273–74). Gabbai’s case is therefore a particularly illustrative example of the power of mental health evidence in England and Wales to render a bodymind “sensible” and hence culpable, for the violence they were subjected to.

## Memory and Mental Health Evidence

This significance of mental health evidence in determining whether a person’s experience represents ‘normal trauma’ or an ‘abnormal diagnosis’ is important here for two reasons. Firstly, it betrays a legal scepticism of all memories of sexual violence where mental health evidence is concerned, as all cases in which either appeals were dismissed or compensation was awarded were corroborated by evidence of ‘trauma’. Secondly, these discursive constructions betray a specific legal construction of the relationship between the ‘mind’ and memories of sexual violence. First, there is the notion of the abnormal ‘mind’, vulnerable to misinterpreting or misrepresenting reality or events. Second, there is the conception of trauma as a truth to be unearthed – the memory as an untouched snapshot waiting for clinical discovery (and verification). In other words, this psychopathological notion of trauma treats sexual violence testimony as real, ‘normal’, and legitimate, and whether someone is ‘abnormal’ and unreliable, or ‘normal’ and truthful, is in need of expert verification (Alcoff and Gray 1993).

### Abnormal Mind and Unreliable Memory

One of the problems with this construction is that applying an ‘abnormal diagnosis’ to an individual bodymind renders them unable to *ever* give reliable evidence concerning sexual violence. In *R v Smith*,^42^ it is written that

The fact of suffering from a severe borderline personality with associated histrionic and dependent features does not by itself necessarily discredit a person’s reliability in all matters… I am sure the complainant should be considered as being capable of giving reliable evidence when she is relatively well, on mental or general subjects. It is more doubtful whether she is capable of doing so accurately when sexual matters are involved.^43^

In another psychiatric report it was “said that the woman’s disorder would involve a failure of normal perception in relation to emotional rather than factual matters… if corroborated, her account of a sexual incident would be credible”.^44^ Such assessments are transparently damning, as both paint the complainant as *incapable* of narrating *any* experience of sexual violence reliably, without corroboration, as these matters are “emotional rather than factual”. This judgment is such that, while she may sincerely believe that she was sexually assaulted, her testimony is inherently unreliable. However, her account was indeed corroborated by both extensive physical medical evidence and a witness, Peter Dawson, who had been with Smith later on that evening when he expressed his fear of being caught: ‘I hope she doesn’t shout rape’.^45^ It is important to note here how significant corroborative evidence is in determining the truth or falsity of an account of sexual violence – particularly as judges still retain discretion as to whether to make a corroboration warning in sexual violence trials (Leahy 2014).

This additionally betrays a legal view of the mind as mechanistic, with ‘mental health’ having a direct bearing on perception and memory. To place this observation in its medico-legal context, the admissibility of evidence of ‘abnormal’ mental health is related to the case of *Toohey v Commissioner of Police of the Metropolis*,^46^ in which the ability of someone with a mental health problem to accurately perceive events was compared to that of a cataract on vision: “it must be allowable to call medical evidence of mental illness which makes a witness incapable of giving reliable evidence, whether through the existence of delusions or otherwise”.^47^ The applicability of the Toohey case to sexual violence trials was ultimately dismissed as outdated in a case concerning sexual abuse of a child.^48^ However, this understanding of mental health remains evident in cases since then: ‘mental health’ is considered to have a direct bearing on individuals’ ability to perceive events, in the way of a cataract on vision.

For example, in one Welsh case, a woman testifying to sexual violence was deemed “manipulative and capable of influencing others to support her allegations”,^49^ and it was argued that a consensual “relationship” had begun when she was 13, when her assailant was 40.^50^ In this case, it is plausible that she was also forced to contend with a personality disorder diagnosis, although either a typo in the transcript or a mistake in the judgment has called it “Dialectic Behaviour Disorder”.^51^ The interpretation of this condition is that she had ‘Dialectic Behaviour Disorder, a condition which is characterised by taking extreme positions’.^52^ Donald Adams had begun an appeal against his convictions for sexual violence, although he died before the appeal went ahead in 2019. The case at the appeal was that that the ‘disordered’ woman “was now, many years after the event, viewing her past relationship with him through that *prism* in a distorted way”.^53^ To reiterate, this argument was advanced in spite of the fact that Adams was 27 years older than this woman, and that their supposedly consensual “relationship” had begun when she was 13. This appeal was resulted in Adams being posthumously acquitted. The idea that someone’s psychiatric diagnosis functions as a *“prism”* of distortion on traumatic memory is directly comparable to the language in Toohey, and the effect of a cataract on vision. As with the Smith case above, it uses a diagnosis to render someone unable to *ever* reliably narrate sexual violence as their memories are considered ‘disordered’ and unreliable (Haaken 1998, 51).

## Traumatic Memory as ‘Snapshot’

The association of an ‘abnormal diagnosis’ with distorted memories of violence can then be contrasted with an understanding of ‘normal’ traumatic memories as veridical snapshots of an experience. Alcoff and Gray (1993) have noted the conception of ‘normal’ sexual trauma as untouched ‘raw experience’ in need of expert verification. On this account, the traumatic memory is pathological – encoded ‘wrongly’, inaccessible to consciousness apart from in flashbacks and dreams. This has been elaborated by several trauma theorists such as US philosopher Susan Brison, who wrote that:

It’s accurate because untouched (like an unretouched photo), not worked over or thought about with the distorting categories of cognition. This apparently gives it privileged epistemological status as a bearer of the truth – as that which, for ethical and political reasons, must be preserved. This preservation, however, comes at the cost of ongoing pathology, and is in conflict with survivor’s goal of psychic recovery (Brison 2002, 70).

Brison’s observation here that traumatic memories must be preserved by not working over the memory through cognition or testimony, is notable in the materials identified for this article – individuals are rewarded for *not* talking about their experience. In Naveed Soroya’s case, it was argued that the woman he assaulted was unreliable, as she mentioned a prior experience of sexual assault in her witness statement to the police. However, there was no record of her telling anyone previously. Thus, the memory of this experience was considered successfully ‘preserved’; a conception that was explicitly verified by the expert psychiatric evaluation, which described the woman as “nice, believable”.^54^ In a related case concerning a man groomed by his PE teacher well into his adulthood, his first disclosure coincided with a psychological breakdown, and when disclosed to his treating mental health facility, was immediately reported to the police.^55^ In both cases, the accounts of the sexual violence produced for trial were only the second time that they had spoken about it on record, and thus the memory had been successfully ‘preserved’ from contamination. This can be contrasted with *R v Gabbai* above, in which multiple prior disclosures of experiences of sexual violence contributed to his acquittal.^56^

The notion of preserving and immediately documenting these initial, pure, forms of disclosure for use as ‘corroborative’ evidence of violence was much more emphatic in the 2020 draft of the Crown Prosecution Service’s guidance on pre-trial therapy published ahead of public consultation. In this document, therapists were repeatedly reminded to respond to any new disclosures by “encourag[ing] the victim to report the offence to the police as soon as possible” (Crown Prosecution Service 2020, 6); and fostering ‘trust’ with people was retooled in service of this: “it is only when a victim develops a sense of trust that they will more fully disclose what has happened” (Crown Prosecution Service 2020, 10). These disclosures were also required to be “fully documented”, in case of a therapist being called as a witness (Crown Prosecution Service 2020, 8). While the newer version has since removed this search for corroborative ‘proof’ so explicitly, the law’s view of traumatic memory as pathological, encoded differently and therefore existing in a different state, is evident in the enduring document on psychological evidence, which states that

Research has shown that under ordinary conditions people with PTSD often have fairly good psychosocial adjustment; however, they do not respond to stress in the same way that other people do. Under pressure (such as at interview, or in court) they may act or feel as if they were being traumatised all over again. This high state of arousal may facilitate memory retrieval and therefore should not necessarily be avoided, although significant explanation and support will be needed. During the attack the survivor was overpowered and helpless. They need to feel that they have control over their life again. Individuals can be helped if they are allowed to make decisions, where possible. Simple techniques such as enabling them to decide if they need a break during testimony can be helpful. This will not only empower them, but will also prevent the compassionate judge rising just at the point when arousal is positively impacting on accessing memories (Crown Prosecution Service 2019, 23).

Literally placing an individual back in a state of trauma is deemed to “facilitate memory retrieval”, and suggested measures are given to preserve this state to ensure it is “positively impacting on accessing memories”. This extract is also particularly interesting for its enlisting of feminist-inspired language – to “empower” the “survivor” to take ‘control’, in order to combat their sense of feeling “overpowered and helpless”. Despite paying lip-service to feminist principles of empowerment and agency, the message of this guidance is clear: people are required to, as stated in another judgment, “submit to the trauma of giving evidence”.^57^

## Conclusion

While the language of trauma has arguably improved understanding of experiences of sexual violence, legal constructions of mental health evidence are soberingly restrictive. The two available discursive constructions identified here can be demarcated along a normal/abnormal binary understanding of mental health. ‘Normal trauma’ is characterised by a subject who is psychologically ‘normal’ before sexual violence, and traumatised and frozen thereafter; reflective of 19th and early 20th century notions of ‘insensibility’ (Bourke 2012). ‘Abnormal diagnosis’ then speaks to the opposite side of this binary: characterised by a ‘sensible’ subject, who is inherently unreliable, whether wittingly or unwittingly. These two discursive constructions betray a legal scepticism of mental health evidence in the context of sexual violence, and a preference for traumatic memories that are successfully ‘preserved’ (because unspoken), rather than ‘contaminated’.

This shows that, in spite of the recent feminist “campaign win” restricting the use of mental health evidence in sexual violence trials, mental health evidence is likely to be considered ‘relevant’ in sexual violence cases, either to establish ‘normal trauma’ or ‘abnormal diagnosis’. This has concerning implications for anyone who has experienced sexual violence and has also sought support for their mental health. It demonstrates the significant gravitational pull of the trauma paradigm for understanding experiences of gendered violence (Sweet 2021), which obscures the legitimacy of individual experiences that do not present in the normal, expected, and traumatised way (Gavey and Schmidt 2011). In addition, rather than finding significant changes in the legal treatment of sexual violence and mental health evidence over time, the analysis demonstrates powerful consistency in these discursive constructions. Even where case law or medico-legal ideas about sexual violence and mental health supposedly change or develop (Bourke 2012), and legal policies are redrafted (Crown Prosecution Service 2022; 2020), the same foundational ideas endure in the bedrock of the legal treatment of sexual violence.^58^ This paper urgently calls for feminists to not be distracted by the recent “campaign win”, but to squarely focus our continued attention on mental health evidence in sexual violence trials, and on the discursive relationship between sexual violence and mental health more broadly.

## Footnotes

1 I use the term ‘bodymind’ in line with critical trauma and disability scholars (Carter 2021).

2 Victims and Prisoners Act 2024, c. 21, s 44 A.

3 *R v Jones & Anor* [2019] EWCA Crim 1570; *re M (An Adult) (Capacity: Consent to Sexual Relations)* [2014] EWCA Civ 37; *R v A* [2014] EWCA Crim 29*9; R v C* [2009] UKHL 42; *R v E* [2011] EWCA Crim 1690; *A Local Authority v TZ* [2013] EWCOP 2322.

4
*R v Turner* [1974] QB 834 at 841.

5
*London Borough of Haringey v FZO* [2020] EWCA Civ 180.

6
*R v Allison* [2006] EWCA Crim 706 at [36].

7
*Supra* n 6 at [22].

8
*Supra* n 6 at [21].

9 *Supra* n 6 at [6].

10 *Lawson v Executor of the Estate of Dawes (Deceased)* [2006] EWHC 2865 (QB) at [42].

11 *R v JD* [2008] EWCA Crim 2557.

12 [2006] EWCA Crim 3120.

13
*Supra* n 12 at [6].

14
*Supra* n 12 at [7].

15
*Supra* n 12 at [14].

16
*Supra* n 12 at [6].

17
*Supra* n 12 at [7].

18
*Supra* n 12 at [14].

19
*Supra* n 12 at [14].

20
*Supra* n 10 at [42].

21
*Supra* n 6 at [6].

22
*R v Allison* [2006] EWCA Crim 706; *Lawson v Executor of the Estate of Dawes (Deceased)* [2006] EWHC 2865 (QB); *R v Soroya* [2006] EWCA Crim 312; *London Borough of Haringey v FZO* [2020] EWCA Civ 180; *DSD & Anor v The Commissioner of Police for the Metropolis* [2014] EWHC 436 (QB).

23
*DSD & Anor v The Commissioner of Police for the Metropolis* [2014] EWHC 436 (QB) at [69].

24
*Supra* n 23 at [71].

25
*Supra* n 23 at [70].

26
*Supra* n 23 at [71].

27
*Supra* n 5.

28
*Supra* n 5 at [85].

29
*R v Gabbai* [2019] EWCA Crim 942 at [38].

30
*Supra* n 29 at [38].

31
*Supra* n 29 at [39].

32
*Supra* n 29 at [40].

33 Supra n 29 at [41].

34 Supra n 29 at [42].

35 Supra n 29 at [59].

36 Youth Justice and Criminal Evidence Act 1999, s 100.

37
*Supra* n 29 at [41].

38
*Supra* n 29.

39
*Supra* n 29 at [15].

40
*R v Gabbai* [2018] Kingston Crown Court at 38–39.

41
*Supra* n 40 at 9.

42 [2002] EWCA Crim 2074.

43
*Supra* n 42 at [21].

44 Supra n 42 at [29].

45 Supra n 42 at [9].

46
*Toohey v Commissioner of Police of the Metropolis* [1965] A.C. 595.

47
*Supra* n 54 at 162.

48
*R v H* [2014] EWCA Crim 1555.

49
*R v Adams* [2019] EWCA Crim 1363 at [13].

50
*Supra* n 49 at [9].

51
*Supra* n 49 at [9].

52
*Supra* n 49 at [9].

53
*Supra* n 49 at [9]; emphasis mine.

54
*Supra* n 12 at [17].

55
*Supra* n 5.

56
*Supra* n 29.

57
*R v Boulton* [2007] EWCA Crim 942 at [13].

58 Supra n 46 and 4.
